# Is Hydrogel an Appropriate Bioadhesive Material for Sutureless Oral Wound Closure?

**DOI:** 10.1002/hsr2.70249

**Published:** 2024-12-09

**Authors:** Huda Moutaz Asmael Al‐Azzawi, Rita Paolini, Antonio Celentano

**Affiliations:** ^1^ Melbourne Dental School The University of Melbourne Carlton Victoria Australia

**Keywords:** bioadhesive, biomimetic, hydrogels, oral wound closure, sutureless oral wound

## Abstract

**Background and Aims:**

An effective surgical adhesive must possess strength, biodegradability, flexibility, non‐toxicity, and the ability to accommodate to tissue movement. However, existing adhesives in the market lack some of these crucial properties. Both synthetic cyanoacrylate and natural fibrin glue have been explored for sutureless oral surgery, but they come with specific limitations. This perspective review aims to explore the novel potential of hydrogels as bioadhesives for wound closure in the oral cavity.

**Methods:**

This review thoroughly examines the properties, applications, and limitations of hydrogels as bioadhesive materials for wound closure within the human body.

**Results:**

We first provide a comprehensive description of materials used for sutureless oral surgery. Next, drawing on our expertise in the field of oral surgery, we propose novel potential applications for hydrogels in oral wound closure. We showed that Hydrogels represent promising bioadhesives in medical field and are undergoing continuous enhancement to expand their applications in wound closure.

**Conclusion:**

Although hydrogels have been utilized in various dental conditions, their potential for closing wounds in the oral cavity remains unexplored.

## 1 Introduction

Instead of conventional suturing for oral surgical and traumatic wounds, a minimally invasive technology utilizing biodegradable adhesive materials can be employed. These materials can retain flap edges in their original position, protect the wound from oral microbes during the healing process, and enhance healing outcomes. This approach reduces operation time and eliminates the need for suture removal, thereby overcoming several disadvantages associated with conventional sutures. These include secondary trauma to tissue caused by needle insertion as well as risk of inflammation, infection, and delay healing. Moreover, it eliminates the risk of needle puncture accidents [1].

Bioadhesion, the phenomenon in which substances, whether natural or synthetic, adhere to biological surfaces. According to their applications, they are grouped into three categories: (A) wound closure, (B) sealing leakage including blood leakage, and (C) immobilization such as wound dressing and drug delivery materials [2]. The requirements of bioadhesives for wound closure differ from those for sealing leakage and so on. Bioadhesive materials for wound closure in the oral cavity should be biocompatible, bioadhesive in wet environment, biodegradable, mechanically strong, easy to apply, and capable of incorporating drugs, cells, and biological factors into the tissue [3]. Till now, only two bioadhesives (cyanoacrylate and fibrin glue) have been evaluated in oral wound closure as an alternative to sutures.

In recent years, hydrogels have gained great interest in the biomedical field due to their outstanding biocompatibility, biodegradability, and high‐water content. Hydrogels are three‐dimensional networks of crosslinked polymers that mimic the natural extracellular matrix (ECM) [4]. Our article provides a comprehensive overview of the current challenges in surgical bioadhesives, focusing on their application in surgical or traumatic wound closure, and proposes hydrogels as a promising solution.

## 2 Bioadhesives Utilized in Oral Wound Closure

### 2.1 Traditional Adhesive: Cyanoacrylate

Cyanoacrylate, a synthetic adhesive, has been evaluated for wound closure in dentistry. Approved by the U.S. Food and Drug Administration in August 1998, cyanoacrylate adhesives polymerize rapidly in the presence of nucleophiles such as anions (e.g., hydroxide, alcoholate and iodide) or weak bases (e.g. alcohol and amine), releasing a slight exothermic effect [5, 6, 7].

Cyanoacrylates are present in various forms depending on their chain length and complexity; these include methyl, ethyl, isoamyl, n‐butyl, isohexyl, and octyl cyanoacrylates [8]. However, it has been observed that the harmful effect decrease as the alkyl chain length increases. Consequently, cyanoacrylate (CA) monomers with short alkoxy carbonyl side chains, such as methyl 2‐cyanoacrylate and ethyl 2‐cyanoacrylate, are no longer used. In contrast, *n*‐butyl‐2‐cyanoacrylate (NBCA) and octyl‐2‐cyanoacrylate (OCA) have gained widespread utilization in clinical practice [9]. There are comprehensive literature reviews on the applications of cyanoacrylates in dentistry [1, 10]. A systematic review and meta‐analysis of six studies involving a total of 250 patients has been published, examining the utilization of cyanoacrylate‐based adhesive and comparing it to silk sutures in third molar surgery. Three studies are split‐mouth design controlled clinical trials, and three are randomized controlled trials (RCTs). They concluded that the utilization of cyanoacrylate adhesive may offer benefits in terms of postoperative pain and swelling reduction [11]. Another systematic review and meta‐analysis, involving eight studies with a total of 440 patients, evaluated the use of cyanoacrylate in third molar surgery and concluded that it promoted better results in pain reduction on the first postoperative day compared to the use of conventional suture [12]. Yet, cyanoacrylate is not used in clinical settings due to its reduced tensile strength (it should not be used in high‐tension areas) [13]. Moreover, cyanoacrylate has disadvantages, including slight heat release, dry surface work, and degradation to produce harmful substances during polymerization [9]. They undergo degradation in the presence of water to produce formaldehyde and cyanoacetate as end products [6].

### 2.2 Fibrin Glue

Fibrin glue (TISSEEL) is a naturally derived adhesive that functions similarly to natural glue, that adhering tissues together. It is an absorbable blood‐derived adhesive, mimicking the final stages of the coagulation cascade, forming a robust clot that seals wound edges. After 2 weeks of application, it physiologically degrades into granulation tissue [14]. A recent systematic review on the utilization of fibrin glue in periodontal surgery included four RCT [15] with a total of 101 patients. This systematic review indicates a low level of evidence for the use of fibrin sealant as an alternative to sutures in periodontal practice. In another study, authors compared fibrin sealant with suturing using 3–0 black silk in third molar extraction surgery through conventional Ward's incision, concluding that fibrin sealant is an effective means of mechanical and biological closure of intraoral wounds [16]. The main limitations of this material are allergic anaphylactic reactions and its inability to address massive bleeding [17]. Additionally, its insufficient adhesion or cohesion strength limits its application, and making it useful only as an adjunct to traditional wound closure [18].

## 3 Hydrogel: A Promising Alternative?

Hydrogels are three‐dimensional polymers of natural or synthetic origin with high water absorbing capacity, resulting in their gel‐like form. Synthesized hydrogels exhibit significant properties such as biocompatibility, mechanical strength, biodegradability, swellability, and stimuli sensitivity [19]. They find a wide range of applications in tissue engineering and regenerative medicine [20]. Natural hydrogels composed of natural polymers such as polysaccharides (chitosan, cellulose, alginate), collagen‐based hydrogels [21] and others. Synthetic hydrogels comprise of synthetic polymers such as polycaprolactone (PCL), poly (ethylene glycol) (PEG), poly (vinyl alcohol) (PVA), and poly (lactic acid) (PLA), or mixtures are also used [19].

Hydrogels can be presented in a solid form or as a liquid that forms gels through various mechanisms, among them cooling or heating. Moreover, they can be manufactured as sheets, matrices, films, and microspheres depending on the method of polymerization [22]. The hydrogel state is achieved through chemical crosslinking via covalent bonds, physical crosslinking through molecular entanglement, hydrogen bonding, hydrophobic interactions, and complexation involving polyelectrolyte interactions [19].

They can be classified depending on the origin, method of preparation, ionic charges, and physical structure [23]. A detailed discussion of their classifications is illustrated in Table 1.

**Table 1:** Overview of hydrogel classifications.

Hydrogels classsification						
Based on origin [24]	Based on method of preparation [25]	Based on cross‐linking (structure) [24, 26]	Based on their reaction with the environment [22, 24]	Based on their degradability [27]	Based on physical properties [25]	Based on ionic charge [24]
Natural hydrogels	Copolymeric (two or more different types of monomers)	chemical hydrogels (cross‐linked by covalent bonds, which are stable and nonreversible)	pH‐responsive	Biodegradable Hydrogels	Smart hydrogels	Non‐ionic or neutral hydrogels (do not have a net electrical charge)
Synthetic hydrogels	Homopolymeric (a single type of monomer)	Physical hydrogels (held together by noncovalent interactions which can be reversible	Temperature‐Responsive	Nonbiodegradable hydrogels	Conventional hydrogels	Cationic (contain positively charged groups)
Hybrid hydrogels	Interpenetrating network (IPN) (two or more polymer networks)	Physical‐chemical hydrogels (hybrid)	Magnetic and electric field responsive	Degradable under Sspecific environmental conditions		Anionic (contain negatively charged groups)
			Pressure responsive			
			Photo‐responsive Antigen responsive			

### 3.1 Applications of Hydrogels in Dentistry

- Dental caries and pulp tissue engineering
  Hydrogel scaffolds incorporated with stem cells and growth factors have shown promise in pulp‐tissue engineering for regenerating the dentin‐pulp complex [28]. Gelatin methacryloyl (GelMA) has been extensively studied for tissue engineering applications, thanks to its beneficial properties, including hydrophilicity, integrin‐binding motifs, and matrix metalloproteinase degradation [29]. Ribeiro et al. developed GelMA hydrogel loaded with chlorhexidine (CHX) for intraoral delivery of CHX, offering an effective approach to treat dental infections. Moreover, GelMA can serve as a scaffold for delivering therapeutic agents into the root canal, allowing gradual release after application [30]. Numerous studies have demonstrated that hydrogels with appropriate ingredients can enhance the antibacterial activity and promote remineralization of the tooth, indicating strong potential for dental caries treatment [31].
- Periodontal diseases
  In dentistry, hydrogels have been explored for drug delivery in periodontal diseases [32], and as scaffolds in periodontal tissue engineering [33]. They are used to deliver drugs to achieve antibacterial effects and to enhance the regeneration of both soft and hard tissues in periodontal pockets. Injectable and temperature‐responsive hydrogels are more commonly used in various studies because their physicochemical characteristics are better suited to the microenvironment of periodontal pocket. Chang et al. integrated naringin into a pH‐sensitive hydrogel with thermogelling properties. This hydrogel composed of carboxymethyl hexanoyl chitosan and β‐GP effectively reduced periodontal bone loss. Furthermore, a lower pH can influence drug delivery by altering the drug carrier structure [34].
- Peri implant diseases
  Hydrogels have been used as coatings on implant surfaces to prevent peri‐implantitis [35]. Hyaluronic acid hydrogel has been widely used to deliver and release bone morphogenetic protein (BMP‐2) to enhance peri‐implant osteogenesis [36].
- Oral cancer and oral mucosal diseases

Hydrogels have been utilized to deliver drugs to oral cancer and oral mucosal diseases [37]. In the application of hydrogel to deliver drugs to oral cancer drug delivery, hydrogels with adhesive or injectable properties are usually used to deliver chemotherapeutic drugs. For example, Shtenberg et al. combined alginate and liposomes in varying proportions to create a hybrid hydrogel with adhesive qualities, aiming to use this system for prolong release of the anticancer drug doxorubicin (DOX) over an extended period of time [38]. Similarly, Tan et al. integrated a metal‐organic framework mixed with thermosensitive hydrogel PLGA‐PEG‐PLGA to develop an injectable system to load anticancer drugs (DOX and celecoxib) for localized of oral cancer treatment [39].

**Figure 1:**
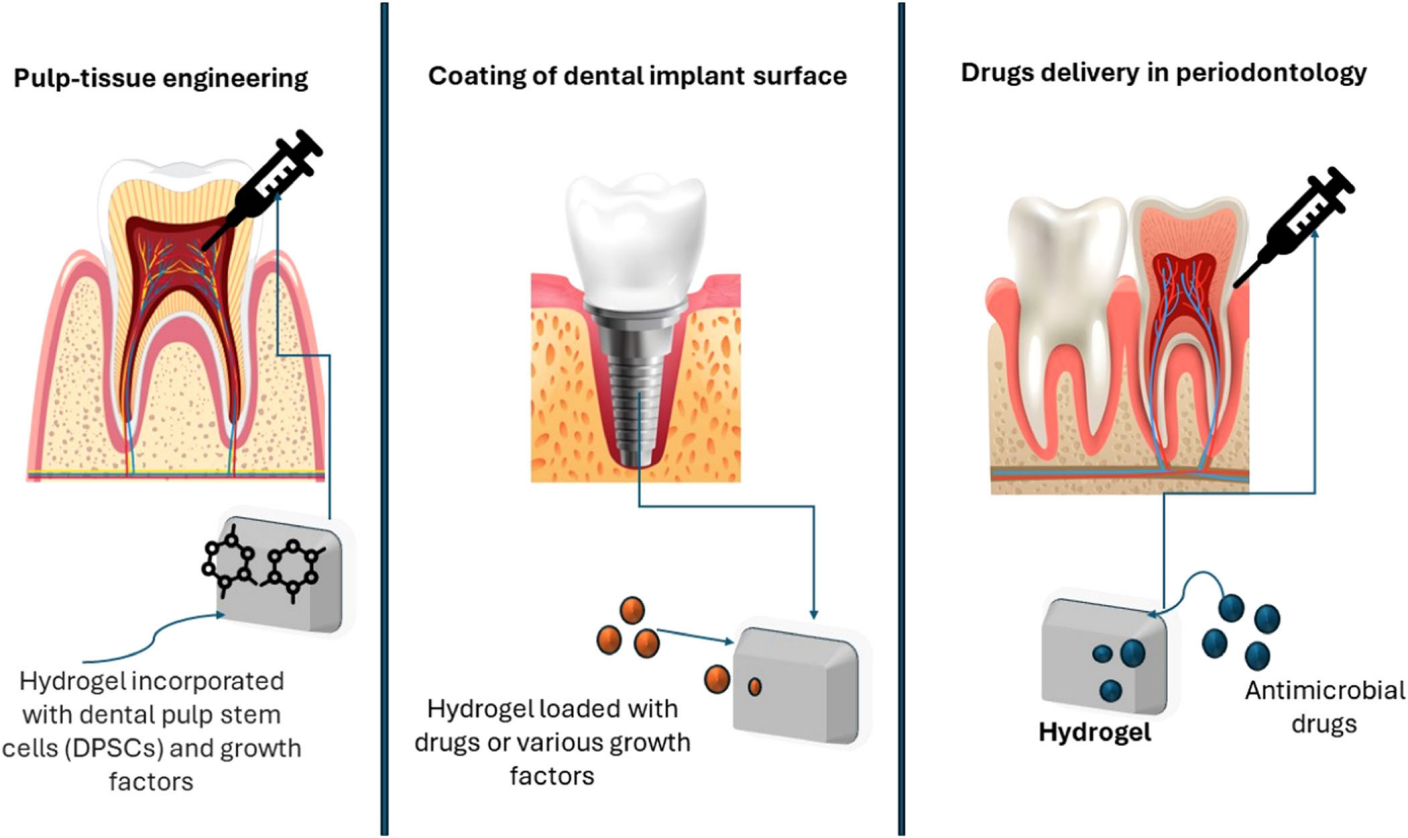
Some hydrogel applications in dentistry; Pictures are under free licence and designed by Freepik then combined by power point. Some components drawn by the authors.

Hydrogels have been used in other oral conditions, such as alveolar Osteitis (dry Socket), osteonecrosis of the Jaw, and in repairing craniomaxillofacial defects [23, 40]. Some applications of hydrogels in dentistry are shown in Figure 1. However, their potential for wound closure in the oral cavity remains unexplored due to various obstacles.

### 3.2 Challenges in Designing Hydrogels for Oral Wound Closure

Wound healing is a physiological process that begins directly after injury. The process of wound healing, progresses through four overlapping stages including, hemostasis, inflammation, proliferation, and remodelling [41]. During the healing process, the bioadhesive should have the ability to form strong interfacial bonds, the ability to have strong cohesive properties, and the ability to remain biocompatible throughout the lifespan of the bioadhesive [42].

**Requirements and challenges of using hydrogels as sutures in the oral cavity are outlined in the points below:**

- Adhesiveness: Hydrogels should maintain strong interfacial bonds with wet oral tissue surfaces. Most bioadhesive utilize more than one type of interaction to maintain these bonds, including chemical crosslinking, diffusion, van der Waals forces, electrostatic interaction, physical interlocking, and micromechanical interactions [42]. However, hydrogel bioadhesives may lose their adhesion in wet environments like in the presence of blood or tissue fluids, hindering interactions between biological surfaces and hydrogels. To overcome this limitation, researchers have adopted the design of new generative hydrogels inspired by mussels, which adhere to wet substances via excreted proteins containing dihydroxyl phenylalanine [18, 43]. Different techniques are employed to boost hydrogel wet adhesion, including incorporating physical or chemical anchoring components into the hydrogel structure, removing interfacial fluids by utilizing hydrophobic residues and dry hydrophilic scaffolds, and deploying multiple adhesive mechanisms concurrently [3]. Researchers have created Mussel‐inspired hydrogel (HA‐catechol/4‐arm‐PEG‐SH), which is made of a catechol‐modified polymer using 4‐arm‐PEG‐SH as a cross‐linker, and it exhibits excellent adhesion in wet environments [44]. Wu et al. utilized a resilient bioadhesive hydrogel for sutureless repair of dural tissues in rodent and porcine system in a wet environment and under supraphysiological pressure. This dural tough adhesive (DTA) consists of two polymer networks: a polyacrylamide network with permanent crosslinking, offering high elasticity, and an alginate polymer network with reversible crosslinking, capable of redistributing mechanical force energy in underlying tissues. Additionally, a highly adhesive layer of chitosan is incorporated into this tough gel. The authors confirmed its biocompatibility over a 4‐week period in a rat craniotomy model [45].
  - Mechanical strength: Hydrogel must have a good cohesive strength and remain stable after it applied to the tissue. While hydrogels are biocompatible and biodegradable polymers, they have limitations in mechanical strength, (stiffness around 10 kPa and toughness less than 10 J m^−2^), which severely restrict their application in continuous load‐bearing applications such as tissue engineering, where tissues usually exhibit high toughness (1000 J m^−2^), high tensile strength (30 MPa), and high stiffness (1 MPa) [46]. Researchers are investigating various mechanisms for fabricating tough hydrogels, which can be categorized into three main groups: (1) homogenous tough hydrogels including tetra‐polyethylene glycol (Tetra‐PEG) hydrogels, click chemistry hydrogels, radiation crosslinked hydrogels, and slide ring hydrogels, (2) energy dissipating hydrogels such as interpenetrating polymer hydrogels (IPNs), double network hydrogels, and fiber‐reinforced hydrogels, and (3) tough hydrogels with multifunctional crosslinker [47]. A stronger hydrogel could be developed by increasing the level of cross‐linking. However, as cross‐linking increases, the hydrogel's ability to stretch decreases, resulting in a more brittle hydrogel. As a result, achieving an optimal level of cross‐linking is crucial to produce a hydrogel that balances both strength and elasticity [48]. Researchers succeeded in creating mechanically tough hydrogels in the last 20 years [49, 50]. Biomimetic hydrogels, designed to mimic the properties, structure, or function of natural biological tissues, often utilize tannic acid (TA) and interpenetrating polymer network (IPN) hydrogels with both covalent and physical crosslinking. This combination provides enhanced cohesion and adhesion [51, 52]. A good hydrogel adhesive should exhibit strong adhesion and mechanical strength when placed on moving tissue, as in the case of oral tissue. This has been shown to be possible, as demonstrated by the utilization of adhesive hydrogel patch (The THMA/PEGDA/SA hydrogel), driven by hydrogen bonds instead of sutures, in a moving area such as joint wound closure [53].
  - Biocompatibility: A bioadhesive should maintain biocompatibility, allowing for wound closure without causing local or systemic reactions in the host and without toxic effects. Many synthetic polymers are biocompatible and nontoxic to the human cells. In one study, the cytotoxicity of hydrogels such as poly (prop acrylic acid), poly glutamic acid, poly (methacrylic acid), poly (acrylic acid), and poly (methacrylic acid) hydrogels were tested on HeLa cells, and these hydrogels demonstrated 90% cell survival [54]. Most polysaccharides are biocompatible, while although many synthetic hydrogels are nontoxic, their cytocompatibility is inferior to that of natural polysaccharides [54].
  - Biodegradability: Another aspect to consider is biodegradability; the hydrogel should have the ability to degrade over time as the tissue heals. Nevertheless, the adhesive needs to maintain its mechanical strength for a period of time (approximately 1 to 2 weeks) to prevent premature rupture of the repaired oral mucosa wound. Moreover, the degradation end products must be non‐cytotoxic and should allow for rapid cellular ingrowth to regenerate functional tissue [42]. Hydrogels degrade through various mechanisms, depending on their chemical composition and environmental conditions (pH and temperature) of oral cavity. Hydrogels may undergo degradation and subsequent elimination from the body through bio‐erosion and bio‐absorption. Biodegradable polymers include hydrophilic, natural, and synthetic polymers [54].

The application of hydrogels in oral cavity wounds closure presents challenges due to the continuous movement of oral muscular tissue and the wet environment resulting from the presence of saliva. These challenges necessitate new scientific investigations and innovative engineering designs of hydrogels. Scientists should specifically design hydrogels for oral wound closure, emphasizing their wet adhesion properties. Moreover, other factors such as the specific wound pH environment and tissue movement can affect the adhesion of such materials. Based on insight from the literature [3], advancements in hydrogel engineering enable their utilization in gastrointestinal wounds with a high pH environment and tissue movement, indicating promising clinical translation in sutureless oral wound closure [3].

## 4 Perspectives and Conclusions

Our study proposes the potential of using hydrogels as an innovative approach for suturing oral wounds. This exploration is motivated by the unique properties of hydrogels, including moisture‐retaining properties, flexibility, adaptability and biocompatibility. Stimuli‐responsive hydrogels, also known as smart hydrogels, are hydrogels that experience significant changes in their swelling behaviour in response to in environmental stimuli such as temperature, pH, and so on [55]. The swelling properties of hydrogels can be beneficial in oral wound closure. Hydrogels can absorb and retain large amounts of water, which helps to maintain a moist environment at the wound site. This is crucial for promoting faster healing and reducing tissue dehydration. Moreover, as hydrogels swell, they can create a cushioning protecting layer over the wound which protects the wound from mechanical stress or irritation. Moreover, the swelling behaviour of hydrogels allows them to act as a carrier for medications. The adoption of hydrogels in oral wound management could transform clinical practices by offering a less invasive, and more comfortable alternative to traditional suturing.

Some hydrogels present promising potential for use as sutures in wet environments. For instance, mussel‐inspired hydrogel such as catechol‐modified hydrogels, which are based on polymers like polyethylene glycol (PEG) or polysaccharides modified with catechol groups, are noteworthy due to their excellent wet adhesion and flexibility properties. On the same instance, a chitosan‐based hydrogel composed of chitosan, a known natural polysaccharide derived from chitin, which is recognized for its biocompatibility, biodegradability, and intrinsic antibacterial properties. Combining these materials with agents such as Catechol‐functionalized chitosan and dibenzaldehyde‐terminated polyethylene glycol (DB‐PEG2000) has created a formulation that has shown potential for sutureless dural closure in preclinical studies [56]. With further advancements and modifications, this approach could be a promising candidate for use in the oral cavity. In a summary, this study provides initial insights into the application of hydrogels for oral suturing, it encourages further research and development in this emerging field. The probability of their adoption depends on various factors, including clinical efficacy, safety profile, cost‐effectiveness, and surgeon preferences.

## Author Contributions

**Huda Moutaz Asmael Al‐Azzawi:** conceptualization, formal analysis, methodology, resources, Validation, visualization, writing–original draft, writing–review and editing. **Rita Paolini:** conceptualization, supervision, visualization, writing–original draft, writing–review and editing. **Antonio Celentano:** conceptualization, methodology, project administration, resources, supervision, validation, visualization, writing–original draft, writing–review and editing. All authors have read and approved the final version of the manuscript.

## Conflicts of Interest

The authors declare no conflicts of interest.

## Transparency Statement

The lead author Antonio Celentano affirms that this manuscript is an honest, accurate, and transparent account of the study being reported; that no important aspects of the study have been omitted; and that any discrepancies from the study as planned (and, if relevant, registered) have been explained.

## Data Availability

The authors confirm that no original data were collected for this perspective study. All relevant literature and materials used for this review are referenced within the article. Antonio Celentano had full access to all of the data in this study and takes complete responsibility for the integrity of the data and the accuracy of the data analysis.

## References

[1] E. Borie , E. Rosas , G. Kuramochi , S. Etcheberry , S. Olate , and B. Weber , “Oral Applications of Cyanoacrylate Adhesives: A Literature Review,” BioMed Research International 2019 (2019): 8217602. 31008113 10.1155/2019/8217602 PMC6441539

[2] W. Duan , X. Bian , and Y. Bu , “Applications of Bioadhesives: A Mini Review,” Frontiers in Bioengineering and Biotechnology 9 (2021): 716035. 34540814 10.3389/fbioe.2021.716035 PMC8446440

[3] X. Hu and M. W. Grinstaff , “Advances in Hydrogel Adhesives for Gastrointestinal Wound Closure and Repair,” Gels 9, no. 4 (2023): 282. 37102894 10.3390/gels9040282 PMC10138019

[4] Y. Ouyang , J. Zhao , and S. Wang , “Multifunctional Hydrogels Based on Chitosan, Hyaluronic Acid and Other Biological Macromolecules for the Treatment of Inflammatory Bowel Disease: A Review,” International Journal of Biological Macromolecules 227 (2023): 505–523. 36495992 10.1016/j.ijbiomac.2022.12.032

[5] S. Mura , E. Fattal , and J. Nicolas , “From Poly(Alkyl Cyanoacrylate) to Squalene as Core Material for the Design of Nanomedicines,” Journal of Drug Targeting 27, no. 5‐6 (2019): 470–501. 30720372 10.1080/1061186X.2019.1579822

[6] P. Sagar , K. Prasad , R. M. Lalitha , and K. Ranganath , “Cyanoacrylate for Intraoral Wound Closure: A Possibility?,” International Journal of Biomaterials 2015 (2015): 165428. 26649041 10.1155/2015/165428 PMC4662987

[7] A. Soni , R. Narula , A. Kumar , M. Parmar , M. Sahore , and M. Chandel , “Comparing Cyanoacrylate Tissue Adhesive and Conventional Subcuticular Skin Sutures for Maxillofacial Incisions—A Prospective Randomized Trial Considering Closure Time, Wound Morbidity, and Cosmetic Outcome,” Journal of Oral and Maxillofacial Surgery 71, no. 12 (2013): 2152.e1‐8. 10.1016/j.joms.2013.08.029 24237777

[8] S. Inal , N. Yılmaz , C. Nisbet , and T. Güvenç , “Biochemical and Histopathological Findings of N‐Butyl‐2‐Cyanoacrylate in Oral Surgery: An Experimental Study,” Oral Surgery, Oral Medicine, Oral Pathology, Oral Radiology, and Endodontology 102, no. 6 (2006): e14–e17. 10.1016/j.tripleo.2006.05.001 17138158

[9] K. Zheng , Q. Gu , D. Zhou , M. Zhou , and L. Zhang , “Recent Progress in Surgical Adhesives for Biomedical Applications,” Smart Materials in Medicine 3 (2022): 41–65.

[10] E. L. Herod , “Cyanoacrylates in Dentistry: A Review of the Literature,” Journal (Canadian Dental Association) 56, no. 4 (1990): 331–334. 2196101

[11] B. Mahardawi , S. Jiaranuchart , S. Rochanavibhata , K. Siriwat , N. Mattheos , and A. Pimkhaokham , “Cyanoacrylate Tissue Adhesive Versus Silk Sutures for Mandibular Third Molar Surgery: A Systematic Review and Meta‐Analysis,” Clinical Oral Investigations 28, no. 3 (2024): 180. 38418796 10.1007/s00784-024-05578-6

[12] M. W. A. Gonçalves , M. R. F. Souza , M. T. Becheleni , E. L. Galvão , E. A. Al‐Moraissi , and S. G. M. Falci , “Does Cyanoacrylate Have the Best Postoperative Outcomes After Third Molar Extractions When Compared to Conventional Sutures? A Systematic Review and Meta‐Analysis,” Heliyon 10, no. 1 (2024): e23058. 38163159 10.1016/j.heliyon.2023.e23058 PMC10755274

[13] A. J. Singer and H. C. Thode, Jr. , “A Review of the Literature on Octylcyanoacrylate Tissue Adhesive,” American Journal of Surgery 187, no. 2 (2004): 238–248. 14769312 10.1016/j.amjsurg.2003.11.017

[14] G. Lopezcarasa‐Hernandez , J. F. Perez‐Vazquez , J. L. Guerrero‐Naranjo , and M. A. Martinez‐Castellanos , “Versatility of Use of Fibrin Glue in Wound Closure and Vitreo‐Retinal Surgery,” International Journal of Retina and Vitreous 7, no. 1 (2021): 33. 33858517 10.1186/s40942-021-00298-5 PMC8050906

[15] M. Mounsif , F. Smouni , and A. Bouziane , “Fibrin Sealant Versus Sutures in Periodontal Surgery: A Systematic Review,” Annals of Medicine & Surgery 76 (2022): 103539. 35495382 10.1016/j.amsu.2022.103539 PMC9052248

[16] M. Gogulanathan , P. Elavenil , A. Gnanam , and V. B. Krishnakumar Raja , “Evaluation of Fibrin Sealant As a Wound Closure Agent in Mandibular Third Molar Surgery—A Prospective, Randomized Controlled Clinical Trial,” International Journal of Oral and Maxillofacial Surgery 44, no. 7 (2015): 871–875. 25721919 10.1016/j.ijom.2015.02.001

[17] B. Jathal , A. Trivedi , and N. Bhavsar , “Use of Fibrin Glue in Periodontal Flap Surgery,” Journal of Indian Society of Periodontology 12, no. 1 (2008): 21–25. 20142939 10.4103/0972-124X.44094 PMC2813549

[18] J. Zhu , H. Zhou , E. M. Gerhard , et al., “Smart Bioadhesives for Wound Healing and Closure,” Bioactive Materials 19 (2023): 360–375. 35574051 10.1016/j.bioactmat.2022.04.020 PMC9062426

[19] S. Bashir , M. Hina , J. Iqbal , et al., “Fundamental Concepts of Hydrogels: Synthesis, Properties, and Their Applications,” Polymers 12, no. 11 (2020): 2702. 33207715 10.3390/polym12112702 PMC7697203

[20] J. Kováč , P. Priščáková , H. Gbelcová , A. Heydari , and S. Žiaran , “Bioadhesive and Injectable Hydrogels and Their Correlation With Mesenchymal Stem Cells Differentiation for Cartilage Repair: A Mini‐Review,” Polymers 15, no. 21 (2023): 4228. 37959908 10.3390/polym15214228 PMC10648146

[21] M. M. Rana and H. De la Hoz Siegler , “Evolution of Hybrid Hydrogels: Next‐Generation Biomaterials for Drug Delivery and Tissue Engineering,” Gels 10, no. 4 (2024): 216. 38667635 10.3390/gels10040216 PMC11049329

[22] E. M. Ahmed , “Hydrogel: Preparation, Characterization, and Applications: A Review,” Journal of Advanced Research 6, no. 2 (2015): 105–121. 25750745 10.1016/j.jare.2013.07.006 PMC4348459

[23] W. Chen , C. Zhang , S. Peng , Y. Lin , and Z. Ye , “Hydrogels in Dental Medicine,” Advanced Therapeutics 7, no. 1 (2024): 2300128.

[24] M. Dhara Ganguly , “Polymer Hydrogels: Classification and Recent Advances,” Journal of Macromolecular Science, Part A 61, no. 5 (2024): 265–288.

[25] F. Ullah , M. B. H. Othman , F. Javed , Z. Ahmad , and H. M. Akil , “Classification, Processing and Application of Hydrogels: A Review,” Materials Science and Engineering: C 57 (2015): 414–433. 26354282 10.1016/j.msec.2015.07.053

[26] M. Bustamante‐Torres , D. Romero‐Fierro , B. Arcentales‐Vera , K. Palomino , H. Magaña , and E. Bucio , “Hydrogels Classification According to the Physical or Chemical Interactions and as Stimuli‐Sensitive Materials,” Gels 7, no. 4 (2021): 182. 34842654 10.3390/gels7040182 PMC8628675

[27] X. Zhang , D. Wu , and C. C. Chu , “Synthesis and Characterization of Partially Biodegradable, Temperature and PH Sensitive Dex‐MA/PNIPAAm Hydrogels,” Biomaterials 25, no. 19 (2004): 4719–4730. 15120518 10.1016/j.biomaterials.2003.11.040

[28] M. M. S. Abbass , A. A. El‐Rashidy , K. M. Sadek , et al., “Hydrogels and Dentin‐Pulp Complex Regeneration: From the Benchtop to Clinical Translation,” Polymers 12, no. 12 (2020): 2935. 33316886 10.3390/polym12122935 PMC7763835

[29] N. Monteiro , G. Thrivikraman , A. Athirasala , et al., “Photopolymerization of Cell‐Laden Gelatin Methacryloyl Hydrogels Using a Dental Curing Light for Regenerative Dentistry,” Dental Materials 34, no. 3 (2018): 389–399. 29199008 10.1016/j.dental.2017.11.020 PMC5818302

[30] J. S. Ribeiro , E. A. F. Bordini , J. A. Ferreira , et al., “Injectable MMP‐Responsive Nanotube‐Modified Gelatin Hydrogel for Dental Infection Ablation,” ACS Applied Materials & Interfaces 12, no. 14 (2020): 16006–16017. 32180395 10.1021/acsami.9b22964 PMC7370252

[31] C. Song , R. Liu , B. Kong , Z. Gu , and G. Chen , “Functional Hydrogels for Treatment of Dental Caries,” Biomedical Technology 5 (2024): 73–81.

[32] B. Wang , H. E. Booij‐Vrieling , E. M. Bronkhorst , et al., “Antimicrobial and Anti‐Inflammatory Thermo‐Reversible Hydrogel for Periodontal Delivery,” Acta Biomaterialia 116 (2020): 259–267. 32937208 10.1016/j.actbio.2020.09.018

[33] D. G. Miranda , S. M. Malmonge , D. M. Campos , N. G. Attik , B. Grosgogeat , and K. Gritsch , “A Chitosan‐Hyaluronic Acid Hydrogel Scaffold for Periodontal Tissue Engineering,” Journal of Biomedical Materials Research Part B: Applied Biomaterials 104, no. 8 (2016): 1691–1702. 26344054 10.1002/jbm.b.33516

[34] P. C. Chang , Y. C. Chao , M. H. Hsiao , et al., “Inhibition of Periodontitis Induction Using a Stimuli‐Responsive Hydrogel Carrying Naringin,” Journal of periodontology 88, no. 2 (2017): 190–196. 27739344 10.1902/jop.2016.160189

[35] W. Boot , D. Gawlitta , P. G. J. Nikkels , et al., “Hyaluronic Acid‐Based Hydrogel Coating Does Not Affect Bone Apposition at the Implant Surface in a Rabbit Model,” Clinical Orthopaedics & Related Research 475, no. 7 (2017): 1911–1919. 28303535 10.1007/s11999-017-5310-0 PMC5449332

[36] H. Pan , J. J. Han , Y.‐D. Park , T. H. Cho , and S. J. Hwang , “Effect of Sustained Release of rhBMP‐2 From Dried and Wet Hyaluronic Acid Hydrogel Carriers Compared With Direct Dip Coating of rhBMP‐2 on Peri‐Implant Osteogenesis of Dental Implants in Canine Mandibles,” Journal of Cranio‐Maxillofacial Surgery 44, no. 2 (2016): 116–125. 26732636 10.1016/j.jcms.2015.11.018

[37] P. Bhardwaj , V. Gota , K. Vishwakarma , et al., “Loco‐Regional Radiosensitizing Nanoparticles‐iIn‐Gel Augments Head and Neck Cancer Chemoradiotherapy,” Journal of Controlled Release 343 (2022): 288–302. 35101477 10.1016/j.jconrel.2022.01.040

[38] Y. Shtenberg , M. Goldfeder , H. Prinz , et al., “Mucoadhesive Alginate Pastes With Embedded Liposomes for Local Oral Drug Delivery,” International Journal of Biological Macromolecules 111 (2018): 62–69. 29292143 10.1016/j.ijbiomac.2017.12.137

[39] G. Tan , Y. Zhong , L. Yang , Y. Jiang , J. Liu , and F. Ren , “A Multifunctional MOF‐Based Nanohybrid as Injectable Implant Platform for Drug Synergistic Oral Cancer Therapy,” Chemical Engineering Journal 390 (2020): 124446.

[40] L. Liu , D. Wu , H. Tu , et al., “Applications of Hydrogels in Drug Delivery for Oral and Maxillofacial Diseases,” Gels 9, no. 2 (2023): 146. 36826316 10.3390/gels9020146 PMC9956178

[41] J. Yang , Z. Wang , X. Liang , W. Wang , and S. Wang , “Multifunctional Polypeptide‐Based Hydrogel Bio‐Adhesives With Pro‐Healing Activities and Their Working Principles,” Advances in Colloid and Interface Science 327 (2024): 103155. 38631096 10.1016/j.cis.2024.103155

[42] R. Pinnaratip , M. S. A. Bhuiyan , K. Meyers , R. M. Rajachar , and B. P. Lee , “Multifunctional Biomedical Adhesives,” Advanced Healthcare Materials 8, no. 11 (2019): e1801568. 30945459 10.1002/adhm.201801568 PMC6636851

[43] H. Yuk , C. E. Varela , C. S. Nabzdyk , et al., “Dry Double‐Sided Tape for Adhesion of Wet Tissues and Devices,” Nature 575, no. 7781 (2019): 169–174. 31666696 10.1038/s41586-019-1710-5

[44] Y. Zhong , X. Zhao , G. Li , D. Zhang , and D. Wang , “Mussel‐Inspired Hydrogels as Tissue Adhesives for Hemostasis With Fast‐Forming and Self‐Healing Properties,” European Polymer Journal 148 (2021): 110361.

[45] K. C. Wu , B. R. Freedman , P. S. Kwon , et al., “A Tough Bioadhesive Hydrogel Supports Sutureless Sealing of the Dural Membrane in Porcine and Ex Vivo Human Tissue,” Science Translational Medicine 16, no. 739 (2024): eadj0616. 38507468 10.1126/scitranslmed.adj0616 PMC11145396

[46] D. L. Taylor and M. In Het Panhuis , “Self‐Healing Hydrogels,” Advanced Materials 28, no. 41 (2016): 9060–9093. 27488822 10.1002/adma.201601613

[47] S. Fuchs , K. Shariati , and M. Ma , “Specialty Tough Hydrogels and Their Biomedical Applications,” Advanced Healthcare Materials 9, no. 2 (2020): e1901396. 31846228 10.1002/adhm.201901396 PMC7586320

[48] X. Li , Y. Zhao , D. Li , G. Zhang , S. Long , and H. Wang , “Hybrid Dual Crosslinked Polyacrylic Acid Hydrogels With Ultrahigh Mechanical Strength, Toughness and Self‐Healing Properties via Soaking Salt Solution,” Polymer 121 (2017): 55–63.

[49] M. Hua , S. Wu , Y. Ma , et al., “Strong Tough Hydrogels Via the Synergy of Freeze‐Casting and Salting Out,” Nature 590, no. 7847 (2021): 594–599. 33627812 10.1038/s41586-021-03212-z

[50] C. Liu , N. Morimoto , L. Jiang , et al., “Tough Hydrogels With Rapid Self‐Reinforcement,” Science 372, no. 6546 (2021): 1078–1081. 34083486 10.1126/science.aaz6694

[51] C. O. Crosby , B. Stern , N. Kalkunte , S. Pedahzur , S. Ramesh , and J. Zoldan , “Interpenetrating Polymer Network Hydrogels as Bioactive Scaffolds for Tissue Engineering,” Reviews in chemical engineering 38, no. 3 (2022): 347–361. 35400772 10.1515/revce-2020-0039 PMC8993131

[52] H. Fan , J. Wang , Q. Zhang , and Z. Jin , “Tannic Acid‐Based Multifunctional Hydrogels With Facile Adjustable Adhesion and Cohesion Contributed by Polyphenol Supramolecular Chemistry,” ACS Omega 2, no. 10 (2017): 6668–6676. 30023527 10.1021/acsomega.7b01067 PMC6045341

[53] H. Liu , X. Hu , W. Li , et al., “A Highly‐Stretchable and Adhesive Hydrogel for Noninvasive Joint Wound Closure Driven by Hydrogen Bonds,” Chemical Engineering Journal 452 (2023): 139368.

[54] Z. Ahmad , S. Salman , S. A. Khan , et al., “Versatility of Hydrogels: From Synthetic Strategies, Classification, and Properties to Biomedical Applications,” Gels 8, no. 3 (2022): 167. 35323280 10.3390/gels8030167 PMC8950628

[55] N. V. Gupta and H. G. Shivakumar , “Investigation of Swelling Behavior and Mechanical Properties of a pH‐Sensitive Superporous Hydrogel Composite,” Iranian Journal of Pharmaceutical Research: IJPR 11, no. 2 (2012): 481–493. 24250471 PMC3832170

[56] G. Ying , W. Fang , H. Zeng , et al., “A Chitosan‐Based Hydrogel Sealant With Effective Closure for Sutureless Dural,” Materials & Design 227 (2023): 111730.

